# CAPTURE and PUMA as Case-Finding Tools in Patients at Risk of COPD—A Multicenter Mexican Experience

**DOI:** 10.3390/jcm14186433

**Published:** 2025-09-12

**Authors:** Arturo Cortes-Telles, Ismael Juarez-de-Dios, Esperanza Figueroa-Hurtado, Diana Lizbeth Ortiz-Farias, Jonathan Alvarez-Pinto, Enrique Olaya-López

**Affiliations:** 1 Clinica de Enfermedades Respiratorias, Hospital Regional de Alta Especialidad de la Peninsula de Yucatan IMSS-Bienestar, Merida 97130, Mexico; esperfh@hotmail.com (E.F.-H.); dianalof16@gmail.com (D.L.O.-F.); 2 Servicio de Medicina Interna, Hospital Regional de Alta Especialidad de la Peninsula de Yucatan IMSS-Bienestar, Merida 97130, Mexico; ismaeljuarez_19@hotmail.com; 3 Servicio de Medicina Interna y Neumologia, Hospital Regional Valentin Gomez Farias ISSSTE, Guadalajara 45100, Mexico; neumo.jap@gmail.com; 4 Hospital Español, Ciudad de Mexico 11870, Mexico; drolaya@yahoo.com.mx

**Keywords:** PUMA, CAPTURE, COPD, sensitivity, specificity, case-finding

## Abstract

**Background/Objectives**: Chronic obstructive pulmonary disease (COPD) is a significant global challenge affecting health systems, economies, and societies. There is a need to implement case-finding strategies to identify patients at risk of COPD in routine clinical practice in low- and middle-income countries where underdiagnosis is high and access to spirometry is limited. PUMA and CAPTURE questionnaires are available for COPD screening; their comparative effectiveness has not been evaluated in Mexican patients. **Methods**: Multicenter, cross-sectional, case-finding study. PUMA and CAPTURE with spirometry test were used in COPD screening campaigns. Adults >40 years of age with current and former smoking, exposure to wood or charcoal, with and without chronic respiratory symptoms were eligible. COPD was confirmed by a post-bronchodilator FEV1/FVC  < 0.70. Sensitivity and specificity were calculated for each questionnaire. Correlations between PUMA and CAPTURE scores and FEV1/FVC were evaluated. Propensity score matching (PSM) was performed for patients with/without COPD. **Results**: In total, 197 subjects were enrolled. COPD was diagnosed in 37.1% of the patients. The cut-off point for PUMA with the best sensitivity (74.0%) and specificity (51.6%) was ≥6. CAPTURE showed cut-off points of ≥3 (sensitivity 76.9%, specificity (40.9%) or ≥4 (sensitivity 61.5%, specificity 72.7%). ROC for PUMA was 0.693 (95% IC 0.618–0.768) and for CAPTURE was 0.714 (95% IC 0.570–0.857). Correlations for PUMA and CAPTURE with FEV1/FVC were −0.390 (*p* < 0.001) and −0.406 (*p* = 0.004), respectively. There were no changes in the cut-off points and correlations after the PSM for PUMA or CAPTURE to identify patients with COPD. **Conclusions**: In Mexican patients, both questionnaires had moderate certainty in the diagnosis of patients at risk for COPD with a spirometric obstructive pattern.

## 1. Introduction

Chronic obstructive pulmonary disease (COPD) is the fourth leading cause of death worldwide. COPD is negatively correlated with quality of life, and it worsens with increasing severity leading to a higher prevalence of subsequent morbidities that result in greater use of healthcare resources [1,2]. The global prevalence is estimated to be 10.3% [GOLD]. However, this condition shows high variability between regions [3,4]. The PLATINO (Project for the Investigation of Obstructive Lung Disease) study showed a wide prevalence in Latin American countries, ranging from 7.8% in Mexico to 19.7% in Uruguay [5].

COPD is characterized by chronic bronchitis and airflow obstruction [3] with progressive deterioration of patient’s lung function [6]. Diagnosis is based on symptoms of airflow obstruction confirmed by spirometry [7]. However, only 26.4% of patients with COPD have had a pulmonary function test [8]. Several studies have consistently reported that the prevalence of COPD is underestimated due to an underdiagnosis or misdiagnosis [8,9,10]. A high proportion occurs in low- and middle-income countries (L-MICs) which have the highest mortality rate of COPD patients (80–85%) [11].

Individuals with underdiagnosed COPD have poor quality of life, suffer from exacerbations, and are at increased risk of death [12]. Among the major known risks are the higher misinterpretation and low awareness of symptoms by the patients and primary care physicians (PCP) [13,14,15]. Most patients are evaluated in primary care (PC) settings, which face several diagnostic challenges due to underestimation of patients’ respiratory symptoms, delayed evaluations, limited resources, and lack of availability and good quality assured spirometry [16].

As PC is the setting in which most of the patients should be diagnosed and treated, it is important to implement case-finding strategies that allow early diagnosis of COPD. PCPs need a simple tool to identify patients at risk of COPD who will eventually undergo spirometry to confirm the diagnosis [17].

The PUMA questionnaire detects at risk patients with COPD with an accuracy of 76% [17] and shows a better performance than CDQ (COPD Diagnostic Questionnaire) and COPS-PS (COPD Population Screener Questionnaire) [18]. However, reports using the PUMA have shown higher variability probably related to specific populations [17]. CAPTURE (COPD Assessment in Primary Care to Identify Undiagnosed Respiratory Disease and Exacerbation Risk) showed a sensitivity of 95.7% and a specificity of 44.4% when peak flow meter is added [19]. Both questionnaires are easy to administer on a PC, but PUMA and CAPTURE have not been used simultaneously in the Mexican Hispanic Population to assess the risk for COPD.

The goal of early detection of COPD [3] is a key challenge in LMICs, where resources are limited in terms of both human and technical support. Diagnostic devices are scarce, and healthcare professionals may not be sufficiently trained to identify COPD risk factors in patients using a variety of questionnaires or by interpreting spirometry reports [7]. Furthermore, there are no national screening or case-finding strategies to help target COPD patients [12]. Patients’ lack of awareness of symptoms is also a contributing factor [12]. National or regional COPD campaigns therefore offer an opportunity to overcome some of these challenges by facilitating interaction between patients and PCPs

As PCPs based COPD diagnoses on patients’ symptoms and physical exam [20], these questionnaires could be a tool to be used by PCPs to better identify patients at risk of COPD. The objective of this study was to determine the sensitivity and specificity of the PUMA and CAPTURE questionnaires in identifying Mexican Hispanic patients at risk of COPD who will benefit from spirometry. Additionally, we aimed to evaluate the accuracy of both questionnaires in identifying patients with a final COPD diagnosis.

## 2. Materials and Methods

### 2.1. Patients

A multicenter, cross-sectional, case-finding study was conducted in three states of Mexico (Yucatan, Jalisco and Ciudad de Mexico) during two World COPD Day campaigns in November 2022 and November 2023. The study was preceded by a preparatory alignment meeting and two meetings to verify its proper progress, where emerging issues were addressed and evidence of discrepancies between centers was resolved during its implementation.

The population was encouraged through social networks, television and radio interviews to attend daytime clinics, and all subjects older than 40 years with risk factors for COPD, including: (a) current or former smokers (at least >5 packs/year), (b) exposure to biomass smoke (wood or coal, cooking or heating) for more than 10 years, (c) with and without respiratory symptoms, were invited to participate. In the first campaign, only the PUMA questionnaire was used, but in the second campaign, both the PUMA and CAPTURE questionnaires were used.

The Ethics Committee of the Hospital Regional de Alta Especialidad de la Peninsula de Yucatan, IMSS-Bienestar, Merida, Mexico, approved this study (Protocol number 2024-020), which was properly registered in accordance with Clause 35 of the Declaration of Helsinki. Moreover, all participants signed and provided oral consent to participate and underwent the pulmonary function test.

Participants received a full explanation of the COPD detection campaign and completed questionnaires (PUMA and CAPTURE) administered by trained staff (certified nurses, nurse practitioners and internal medicine residents). In addition, other socio-demographic variables were collected: age, sex (male/female), self-reported comorbidities, tobacco exposure (current, ex-smoker including pack-years), biomass exposure (exposure duration, hours per year).

Patients with previous diagnosis and treatment of asthma and/or COPD were excluded. Patients with an acute respiratory infection in the previous 4 weeks and those participants without an acceptable spirometry test were also excluded.

### 2.2. Pulmonary Function Testing

All patients underwent post-bronchodilator (post-BD) spirometry, based on the 2019 ATS/ERS technical standardization criteria for acceptability and reproducibility [21]. Spirometry measurements were performed by trained and certified respiratory technicians using mobile spirometers (Easy One spirometer, ndd Medical Technologies, Inc. Technoparkstrasse 1, CH-8005 Zurich, Switzerland) that were calibrated before the session. Spirometry was performed before and after 400 mcg of salbutamol (post-BD) in each subject interviewed. The reports were reviewed by a pulmonologist, and the results were communicated to the participants. All patients with a confirmed diagnosis of COPD were discharged to an outpatient clinic at each site.

### 2.3. COPD Definition

COPD was diagnosed based on the GOLD guidelines [3] with post-BD FEV1/FVC  <  0.70, with the lower limit of normal of FEV1/FVC also considered to strengthen the GOLD diagnosis.

### 2.4. Questionnaires

PUMA is a pre-screening questionnaire to identify patients at risk for COPD. PUMA assesses risk factors (gender, age, smoking pack-years) and respiratory symptoms (dyspnea, sputum production, cough) and has been shown to be a reliable tool for selecting patients at risk for COPD to undergo spirometry [17]. The score ranges from 0 to 9 (higher scores indicate higher risk of COPD) with 0–2 points assigned for each category [17]. CAPTURE is a screening tool to identify patients at risk of having undiagnosed and clinically significant COPD. A positive screening result is defined as (1) a CAPTURE questionnaire score of 5 or 6 or (2) a questionnaire score of 2, 3, or 4 together with a peak expiratory flow (PEF) rate of less than 250 L/min for woman or less than 350 L/min for men [19].

Neither PUMA nor CAPTURE questionnaires require translation as both have been validated in Spanish versions [17,22].

### 2.5. Patient and Public Involvement Statement

Patients were not involved in the development of the research question, study design, outcome measurements or dissemination of the results. However, we conducted the study in accordance with ethical principles and regulations to ensure patient confidentiality and privacy. All patients who were identified as new COPD cases were referred to their clinical facilities to initiate treatment and follow-up.

### 2.6. Statistical Analysis

For quantitative variables, the Kolmogorov test was used, and a non-parametric distribution was identified, so the data are presented as median and interquartile range (IQR). Frequencies and percentages were used for qualitative variables. The Mann–Whitney U test was used to compare quantitative variables, and Chi2 was used for the qualitative variables. The Spearman test was used to calculate the correlation between spirometry results and PUMA and CAPTURE test scores. A linear regression analysis was performed to determine the effect the impact of PUMA and CAPTURE score on FEV1/FVC, adjusted for age, sex, presence of comorbidities, asthma, diabetes, hypertension, and cardiovascular conditions. Factors with a value of *p* < 0.20 were included in the univariate analyses using the Wald test, presented as odds ratios (OR) with the corresponding 95% confidence intervals (CI). The construction of the statistical model was guided by the Akaike criterion and the generality of the results. A *p*-value of <0.05 was considered statistically significant.

Sensitivity and specificity analyses were performed to determine the cut-off points of the PUMA and CAPTURE questionnaires in diagnosing COPD. The overall accuracy was analyzed using a receiver operator characteristic (ROC) curve and the results were reported according to the area under the curve (AUC) values (95% CI). Spirometry results and COPD diagnostic results were compared according to the GOLD classification. The sensitivity and specificity of both questionnaires were assessed using the pre-BD FEV1/FVC values and using the LLN (z-score < −1.645) values to determine obstruction.

#### 2.6.1. Propensity Score (PS) Matching

To minimize the effect of bias introduced by the covariate’s comorbidities, hypertension, diabetes, cardiovascular disease, asthma, age and sex, patient matching was performed using the K-nearest neighbors’ algorithm of the propensity score calculated for these covariates using the psmpy 0.3.13 library for Python v3.11.9. Qualitative variables were tested with χ^2^ test and quantitative variables with the Kolmogorov–Smirnov test and the grouped permutation of the distance χ^2^ after matching.

Statistical significance was considered at *p* < 0.05. The statistical package STATA v.16 (College Station, TX, USA) was used for the analysis.

#### 2.6.2. Sample Estimation

To estimate the minimum number of subjects needed to differentiate between patients with and without COPD, a post hoc calculation was performed. This estimation was based on the previously reported sensitivity and specificity of the CAPTURE [19,23,24,25] and PUMA [17,18,25] questionnaires. However, these values varied significantly depending on the population to which they were applied. Therefore, we used the average sensitivity and specificity values: CAPTURE 72.1% (IC95%: 60.9–82.4%) and 76.14% (IC95%: 67.3–83.0%), respectively, and PUMA 77.9% (IC95%: 66.9–86.9%) and 64.1% (IC95%: 54.6–72.2%), respectively. The statistical parameters considered were: Z_α_ = 1.9600, Z_β_ = 0.8416, V_0_ = Ψ = S_0_ + S_1_ − (2 ∗ S_0_ ∗ P(T_1_− | T_0_−)), V_A_ = Ψ − (S_1_ − S_0_)^2^, A = Z_α_ ∗ √V_0_, B = Z_β_ ∗ √V_A_, C = (S_1_ − S_0_)^2^, N = (A + B)^2^/C [26,27]. The number of subjects for sensitivity was 133 and for specificity was 63. The total number of subjects was estimated to be 196.

## 3. Results

In total, 197 subjects were enrolled in this study. Male gender was slightly more prevalent (58.4%) and 70.6% were >60 years old. Up to 83.8% had comorbidities and systemic hypertension was the most common (45.7%). Most of the included patients were either current or former smokers (91.4%). An obstructive spirometry pattern was identified in 37.1% of the subjects (confirmed COPD population) (Supplementary Table S1).

A comparison was made between groups (patients with and without COPD). Patients with COPD were older (72 years vs. 64 years, *p* < 0.001), had a higher prevalence of self-reported asthma (9.6% vs. 1.6%, *p* = 0.014) and a longer smoking history (33 years vs. 21 years, *p* = 0.001). At diagnosis, patients with COPD had mild to moderate disease based on FEV1 z-score severity and percent predicted values. There were statistically significant differences in both questionnaires (PUMA 7 vs. 5 points, *p* < 0.001; CAPTURE 4 vs. 3, *p* = 0.009) between patients with and without COPD (Supplementary Table S1).

### 3.1. Accuracy of PUMA and CAPTURE to Identify Patients with High Risk of COPD

The cut-off point for PUMA with the best balance of sensitivity (74.0%) and specificity (51.6%) was ≥6. CAPTURE showed cut-off points of ≥3 (sensitivity 76.9%, specificity 40.9%) or ≥4 (sensitivity 61.5%, specificity 72.7%) (Table 1).

**Figure 1:**
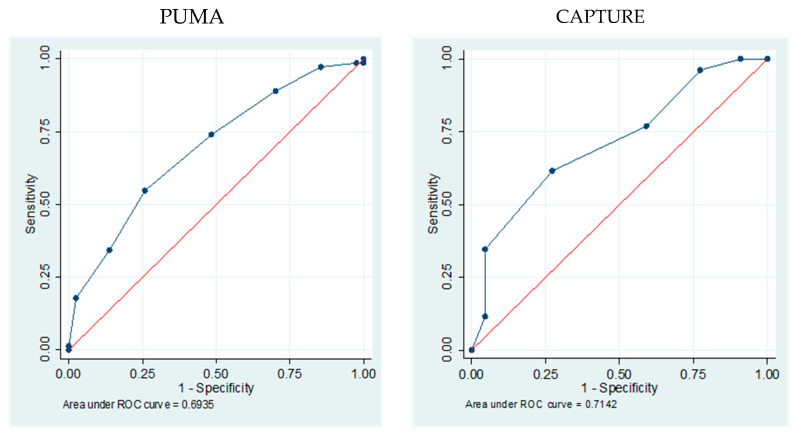
Area under the receiver operating characteristic (ROC) Curve for PUMA and CAPTURE Questionnaires in the screening of COPD among all included patients.

The area under ROC curve for the PUMA (0.693, 95%IC 0.618–0.768) and CAPTURE (0.714, 95%IC 0.570–0.857) questionnaires showed that both questionnaires had moderate certainty in predicting patients at risk of COPD (Figure 1).

The Spearman correlations for PUMA and CAPTURE questionnaires for FEV1 (L) were −0.316 (*p* < 0.001) and −0.401 (*p* < 0.004); and for FVC (L) were −0.181 (*p* < 0.010) and −0.279 (*p* = 0.054); and for FEV1/FVC ratio were −0.390 (*p* < 0.001) and −0.406 (*p* = 0.004), respectively, all with statistical significance (Supplementary Table S2).

After performing the PS matching, the subjects with the most similar propensity scores were selected (Supplementary Figure S1). All qualitative and quantitative variables have a *p* > 0.05 value after PS matching. Comparisons between patients with and without COPD are shown in Table 2. There were differences in age (72 vs. 67 years, *p* = 0.030) and in smoking history (33 vs. 21 years, *p* = 0.008).

An exploratory analysis was performed using pre-BD FEV1/FVC. The cut-off points for PUMA that offered the best balance were ≥6 (with a sensitivity of 48.4% and a specificity of 26.0%) and ≥7 (with a sensitivity of 25.8% and a specificity of 45.2%). CAPTURE showed a cut-off point of ≥3 (sensitivity 59.1%, specificity 23.1%). The area under the ROC curve was 0.30 (95% CI 0.23, 0.38) for PUMA and 0.28 (95% CI 0.14, 0.42) for CAPTURE (Supplementary Table S6).

A supplementary analysis was performed using LLN (z-score < −1.645) to define obstruction. The cut-off points for PUMA that offered the best balance were ≥6 (sensitivity: 76.5%; specificity: 48.6%) and ≥7 (sensitivity: 60.8%; specificity: 71.9%). CAPTURE showed a cut-off point of ≥4 (sensitivity 55.0%, specificity 60.7%). The area under the ROC curve was 0.69 (95% CI 0.61, 0.77) for PUMA and 0.65 (95% CI 0.61, 0.77) for CAPTURE (Supplementary Table S7).

**Figure 2:**
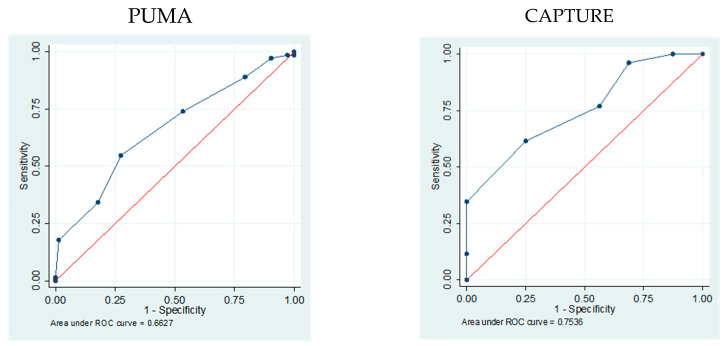
Area under the receiver operating characteristic (ROC) curve for the PUMA and CAPTURE after PS matching patients with and without COPD.

The area under the ROC curve for PUMA and CAPTURE after PS matching showed statistical significance in both questionnaires (*p* < 0.001). The AUC for PUMA was 0.66 (95% IC 0.576–0.749), while for CAPTURE it was 0.75 (95% IC 0.611 and 0.895) (Figure 2).

After PS matching, the cut-off point for PUMA in patients with a confirmed diagnosis of COPD was ≥6 (sensitivity 73.9% and specificity 46.6%) and for CAPTURE was ≥4 (sensitivity of 61.5% and specificity of 75%) (Table 3).

The Spearman correlations for PUMA and CAPTURE questionnaires after PS matching were −0.271 (*p* < 0.001) and −0.393 (*p* < 0.009) FEV1(L); −0.133 (*p* = 0.108) and −0.240 (*p* = 0.124) for FVC (L); and −0.276 (*p* < 0.001) and −0.436 (*p* = 0.003) for FEV1/FVC ratio. There were no statistically significant differences for FVC(L) (Supplementary Table S3).

### 3.2. Linear Regression Analysis (LRA)

After the LRA the variables that showed the most significant association with FEV1/FVC were the total score of both questionnaires with coefficients of −0.023 (95%IC −0.034, −0.012) for PUMA and −0.031 (95%IC −0.056, −0.006) for CAPTURE with *p* values of <0.001 and 0.014, respectively (Supplementary Table S4). PUMA had a *p* = 0.0004 for the model, with an R^2^ = 0.2222 (adjusted R^2^ = 0.1891) without collinearity or multicollinearity (FIV = 1.24). CAPTURE had a *p* = 0. 0327 for the model with an R^2^ = 0.3247 (adjusted R^2^ = 0.1862) without collinearity or multicollinearity (FIV = 1.57). Residue plots confirmed the linearity and normality of the models (Supplementary Figure S2).

Once PS matching, LRA questionnaire scores showed a significant inverse association with FEV1/FVC with coefficients of −0.028 (95%IC −0.041–0.015) for PUMA and −0.037 (95%IC −0.058–0.009) for CAPTURE with *p* values of <0.001 and 0.009, respectively (Supplementary Table S5). PUMA had a *p* = 0.0004 for the model, with an R^2^ = 0.1839 (adjusted R^2^ 0.1363) without collinearity or multicollinearity (FIV = 1.28). CAPTURE had a *p* = 0. 0327 for the model with a R^2^ = 0.3737 (adjusted R^2^ 0.2219) without collinearity or multicollinearity (FIV = 1.68). Residue plots confirmed the linearity and normality of the models (Supplementary Figure S3).

PUMA and CAPTURE scores showed a moderate negative correlation with FEV1/FVC that was maintained after PS matching; none of the other variables showed collinearity.

## 4. Discussion

COPD is a leading cause of significant morbidity and mortality worldwide. Its prevalence is expected to increase due to an aging demographic. Despite its high prevalence, COPD is severely underdiagnosed, especially in L-MICs [7,28] due to the diagnosis being made based on the symptoms and physical examination of the patient, although GOLD guidelines recommend the use of spirometry as the gold standard to confirm the diagnosis. This is because access to spirometry in L-MICs is limited, especially in primary care settings [7] and PCPs do not know how to perform or interpret the test [20].

One proposal has been to identify patients at risk of COPD in primary care using peak flow meters, but this strategy seems unrealistic due to the requirements of supplies, staff time and strict monitoring to obtain accurate results [29]. Alternatively, some questionnaires have been developed to support the screening of patients at risk of COPD. PUMA and CAPTURE are found within these questionnaires, and they are easy-to-implement tools able to identify patients at risk of COPD, but the available results have shown heterogeneity according to the population studied [28,30].

In this study, we evaluated both questionnaires in a Mexican Hispanic population of adults with risk factors for COPD. These tools allowed the identification of COPD in 37.1% of the subjects which was confirmed with spirometry.

The best cutoff-point for PUMA to identify patients at risk for COPD and subsequently confirmed by spirometry was ≥6 points (sensitivity 74.0% and specificity 51.6%), with an area under the ROC curve of 0.69; although after point-by-point analysis, the higher sensitivity reflected a cut-off point of ≥5 (89% but a very low specificity 29.8%). López Varela [17] reported a similar cut-off point using PUMA in a heterogeneous cohort of Hispanic patients (≥6 points, sensitivity 69.9%, specificity of 62.1%, area under the ROC curve 0.70) and Aug-Doung et al. [18] also found a higher sensitivity of PUMA using a cut-off score of ≥5 (sensitivity 91.2%) but a lower specificity (42.6%). Our results, although slightly different from those of other authors, confirm that cut-off points of ≥5 or ≥6 of PUMA could be used with moderate accuracy to identify Mexican Hispanic patients at risk for COPD.

For CAPTURE, the best cut-off point was ≥4 (sensitivity 61.5%, specificity 72.7%, area under the ROC curve 0.71). Our results showed a lower performance compared to those reported by Martinez et al. [19], with a sensitivity of 95.7% and specificity of 67.8%. However, its higher sensitivity was associated with the concomitant evaluation of PEF which was not used in our cohort. Nevertheless, our data suggests that with a cut-off point ≥4, CAPTURE has a moderate certainty to discriminate patients who would benefit from spirometry to confirm the diagnosis of COPD.

Using pre-BD FEV1/FVC, the cut-off points with best balance for PUMA were ≥6 (sensitivity of 48.4%, specificity of 26.0%) and ≥7 (sensitivity of 25.8%, specificity of 45.2%). CAPTURE showed a cut-off point of ≥3 (sensitivity 59.1%, specificity 23.1%). These outcomes suggest that both questionnaires have low discriminatory power to identify patients at risk of COPD and that they cannot be used with a simplified lung function test to confirm a COPD diagnosis [31].

A supplementary analysis using LLN (z-score < −1.645) revealed that the optimal cut-off points for PUMA were ≥6 (sensitivity 76.5%, specificity 48.6%) and ≥7 (sensitivity 60.8%, specificity 71.9%). CAPTURE showed a cut-off point of ≥4 (sensitivity 55.0%, specificity 60.7%). The moderate accuracy of both questionnaires suggests that age and sex do not impact their ability to identify patients at risk of COPD [31].

Even with the low–moderate statistically significant negative association between PUMA and CAPTURE and FEV1/FVC (−0.390 and −0.406, respectively), there is a greater possibility of confirming the COPD diagnosis as the scores of the questionnaires are higher. Both instruments could be suitable to be used as a case-finding strategy focused on patients at risk of COPD, mainly in primary care.

The results with both questionnaires in patients with and without a confirmed diagnosis of COPD and after a PS matching, also showed that PUMA (score ≥ 6) and CAPTURE (score ≥ 4) have a moderate accuracy (0.66 and 0.75, respectively) to identify patients at risk of COPD. LRA indicated that the higher the score on either questionnaire, the more likely it was to identify an obstructive spirometry pattern, as there was a mild to moderate inversely proportional association with FEV1/FVC or FEV1. These results were independent of confounding variables such as age, gender, presence of comorbidities such as diabetes, arterial hypertension or cardiovascular disease.

Differences in sensitivity and specificity compared to other authors [7,19,23] can be explained by the proportion of patients reporting respiratory symptoms that ultimately showed an obstructive spirometry pattern [23]. The frequency is much higher than that reported by Martinez et al. [23] and Ho et al. [7] (7% and 30%, respectively), supporting the need to increase the awareness and detection of COPD in L-MICs. As access to spirometry is limited in these countries, the use of questionnaires such as PUMA and CAPTURE will provide an inexpensive means of early identification of those patients requiring spirometry to confirm COPD.

These results suggest that the implementation of either questionnaire may help PCPs to better assess patients at risk of COPD instead of establishing the diagnosis only with clinical symptoms and physical exam [20].

Our study is not free of limitations including the number of subjects evaluated, as we did not have a formal sample size calculation, but we screened patients who attended COPD campaigns. Despite the reduction in the number of cases with the CAPTURE questionnaire (from 48 to 42) for the purposes of the PSM, the accuracy of the sensitivity and specificity indicators and the 95% CI were not compromised and confirmed the findings of the sample universe for CAPTURE. Second, we could not evaluate PEF simultaneously. Third, these subjects might be more aware of the presence of respiratory symptoms and therefore seek medical advice. Fourth, because we included only the Mexican Hispanic population, the results may not be generalizable to other Hispanic populations.

## 5. Conclusions

Despite its high prevalence, COPD is often misdiagnosed in populations in low- and middle-income countries (LMICs), as our results showed. One contributing factor is that no tools are used to assist with the diagnosis of patients at risk of COPD; screening is usually based on symptoms and clinical data. Our findings support the use of the PUMA and CAPTURE questionnaires for screening these patients. Both tools are moderately accurate in identifying COPD and are not affected by age or gender. These low-cost instruments can be easily used in primary care settings or with open populations, ensuring that spirometry is only performed on preselected patients and contributing to the more rational use of healthcare resources. This study will be implemented with the launch of a national registry with the aim of presenting an epidemiological study on the incidence and prevalence of COPD in Mexico.

Further analysis should be carried out in LMIC populations, where factors such as limited education and language proficiency may play a role.

## Figures and Tables

**Table 1:** Sensitivity and specificity of the PUMA and CAPTURE questionnaires with different cut-off points.

Score	Sensitivity (%)	Specificity (%)	Correct COPD Classification (%)	LR+	LR−
PUMA (n = 197)					
≥5	89.0	29.8	51.8	1.52	0.50
≥6	74.0	51.6	59.9	2.12	0.60
≥7	54.8	74.2	67.0	2.49	0.76
CAPTURE (n = 48)					
≥2	96.1	22.7	62.5	1.24	0.16
≥3	76.9	40.9	60.4	1.30	0.56
≥4	61.5	72.7	66.6	2.25	0.58

**Table 2:** Comparisons groups with and without COPD before and after propensity score (PS) matching.

	Before PS Matching (n = 197)			After PS Matching (n = 146)		
Characteristics	Without COPD (n = 124)	with COPD (n = 73)	*p*	Without COPD (n = 73)	with COPD (n = 73)	*p*
Age, years. Median (IQR)	64 (57, 70)	72 (63, 79)	<0.001	67 (61, 74)	72 (63, 79)	0.030
Age, years; frequency (%)			0.177			0.913
40–49	11 (8.9)	3 (4.1)		4 (5.5)	3 (4.1)	
50–59	31 (25.0)	13 (17.8)		12 (16.4)	13 (17.8)	
>60	82 (66.1)	78.1 (78.1)		57 (78.1)	57 (78.1)	
Gender, frequency (%)			0.475			0.864
Female	54 (43.6)	28 (38.4)		27 (37.0)	28 (38.4)	
Male	70 (56.4)	45 (61.6)		46 (63.0)	45 (61.6)	
Comorbidities, frequency (%)	104 (83.8)	61 (83.6)	0.955	55 (75.3)	61 (83.6)	0.219
Arterial hypertension, frequency (%)	55 (44.4)	35 (47.9)	0.625	29 (39.7)	35 (47.9)	0.317
Diabetes Mellitus 2, frequency (%)	38 (30.6)	16 (21.9)	0.185	11 (15.1)	16 (21.9)	0.286
Cardiovascular, frequency (%)	18 (14.5)	16 (21.9)	0.184	13 (17.8)	16 (21.9)	0.534
Asthma, frequency (%)	2 (1.6)	7 (9.6)	0.014	2 (2.7)	7 (9.6)	0.085
Smoking, frequency (%)	111 (89.5)	69 (94.5)	0.227	65 (89.0)	69 (94.5)	0.228
History of smoking, years; Median (IQR)	21 (15, 40)	33 (20, 43)	0.001	20 (15, 40)	33 (20, 43)	0.008
Biomass, frequency (%)	21 (16.9)	7 (9.6)	0.154	13 (17.8)	7 (9.6)	0.149
FEV1, liters; Median (IQR)	2.15 (1.70, 2.71)	1.34 (1.01, 1.78)	<0.001	2.19 (1.75, 2.66)	1.34 (1.01, 1.78)	<0.001
FEV1, %; Median (IQR)	84.0 (73.0, 95.8)	61.0 (47.0, 70.0)	<0.001	84.0 (75.0, 100)	61.0 (47.0, 70.0)	<0.001
FEV1 Z Score, Median (IQR)	−0.91 (−1.61, 0.0)	−2.33 (−2.92, −1.64)	<0.001	−0.77 (−1.50, 0.10)	−2.33 (−2.92, −1.64)	<0.001
FVC, liters; Median (IQR)	2.79 (2.05, 3.40)	2.40 (1.70, 3.23)	0.021	2.91 (2.11, 3.38)	2.40 (1.70, 3.23)	0.026
FVC, %; Median (IQR)	82.0 (72.0, 92.4)	78.0 (59.0, 89.0)	0.023	84.0 (76.0, 94.0)	78.0 (59.0, 89.0)	0.013
FVC Z Score, Median (IQR)	−1.25 (−1.85, −0.47)	−1.46 (−2.13, −0.71)	0.188	−1.21 (−1.71, −0.31)	−1.46 (−2.13, −0.71)	0.076
Relation FEV1/FVC, Median (IQR)	0.78 (0.74, 0.82)	0.60 (0.52, 0.65)	<0.001	0.77 (0.74, 0.81)	0.60 (0.52, 0.65)	<0.001
PUMA Score	5 (4, 7)	7 (5, 8)	<0.001	6 (5, 7)	7 (5, 8)	<0.001
CAPTURE Score	3 (2, 4)	4 (3, 5)	0.009	3 (0, 3.5)	4 (3, 5)	0.005

**Table 3:** Sensitivity and specificity of the PUMA (n = 73) and CAPTURE (n = 42) questionnaires with different cut-off points (n = 73) after PS matching.

	Sensitivity (%)	Specificity (%)	Correct Classification (%)	LR+	LR−
PUMA score					
≥5	89.0	20.5	54.7	1.12	0.53
≥6	73.9	46.6	60.3	1.38	0.55
≥7	54.8	72.6	63.7	2.00	0.62
CAPTURE score					
≥2	96.1	31.2	71.4	1.39	0.12
≥3	76.9	43.7	64.2	1.36	0.52
≥4	61.5	75.0	66.6	2.46	0.51

## Data Availability

Derived data supporting the findings of this research are available from the corresponding author on request.

## Supplementary Materials

The following supporting information can be downloaded at https://www.mdpi.com/article/10.3390/jcm14186433/s1, Table S1: Comparison of general characteristics of total population and patients with and without COPD after spirometry confirmation. Table S2: Spearman correlation between PUMA, CAPTURE, and spirometry parameters before PS matching. Figure S1: Analysis of variables for propensity score matching. Table S3: Spearman correlation between PUMA, CAPTURE and spirometry parameters after PS matching. Table S4: Linear regression to identify the association of the FEV1/FVC index with the PUMA and CAPTURE scores before PS matching. Figure S2: Residual charts to assess linearity and normality for the LRA for PUMA and CAPTURE before PS matching. Table S5: Linear regression to identify the association of FEV1/FVC index with PUMA and CAPTURE scores after PS matching. Figure S3: Residual charts to assess linearity and normality for the LRA for PUMA and CAPTURE after PS matching. Table S6: Sensitivity and specificity of the PUMA questionnaire with different cut-off points (n = 197) using pre-BD FEV1/FVC with a fixed cut-off point of 0.70 (70%). Table S7: Sensitivity and specificity of the PUMA questionnaire with different cut-off points (n = 197) using LLN (z-score < −1.645) to define obstruction.

## Abbreviations

The following abbreviations are used in this manuscript:

ATS/ERS	American Thoracic Society/European Respiratory Society
AUC	area under the curve
CAPTURE	COPD Assessment in Primary Care to Identify Undiagnosed Respiratory Disease and Exacerbation Risk
CDQ	COPD Diagnostic Questionnaire
CI	confidence intervals
COPD	Chronic obstructive pulmonary disease
COPS-PS	COPD Population Screener Questionnaire
FEV1	Forced Expiratory Volume in 1 s
FEV1/FVC	ratio by dividing FEV1 by FV
FVC	Forced Vital Capacity
IQR	interquartile range
L-MICs	low- and middle-income countries
ORs	odds ratios
PC	primary care
PCP	primary care physicians
post-BD	post-bronchodilator
PSM	Propensity score matching
ROC	receiver operator characteristics
