# Supplementary material for: CAPTURE and PUMA as Case-Finding Tools in Patients at Risk of COPD—A Multicenter Mexican Experience
Source: J Clin Med. 2025 Sep 12;14(18):6433. doi: 10.3390/jcm14186433 (PMC12470894; doi:10.3390/jcm14186433)
Supplement: Supplementary file 1 [file jcm-14-06433-s001.zip › jcm-3761541-supplementary.pdf]

ONLINE SUPPLEMENT

CAPTURE and PUMA as Case-Finding Tools in Patients At Risk of COPD—A Multicenter Mexican Experience

Arturo Cortes-Telles [1,*], Ismael Juarez-de-Dios [2], Esperanza Figueroa-Hurtado [1], Diana Lizbeth Ortiz-Farias [1],
Jonathan Alvarez-Pinto [3] and Enrique Olaya-López [4]

1 Clinica de Enfermedades Respiratorias, Hospital Regional de Alta Especialidad de la Peninsula de Yucatan IMSS-Bienestar, Merida 97130, Mexico; esperfh@hotmail.com (E.F.-H.); dianalof16@gmail.com (D.L.O.-F.)
2 Servicio de Medicina Interna, Hospital Regional de Alta Especialidad de la Peninsula de Yucatan
IMSS-Bienestar, Merida 97130, Mexico; ismaeljuarez_19@hotmail.com
3 Servicio de Medicina Interna y Neumologia, Hospital Regional Valentin Gomez Farias ISSSTE,
Guadalajara 45100, Mexico; neumo.jap@gmail.com
4 Hospital Español, Ciudad de Mexico 11870, Mexico; drolaya@yahoo.com.mx
* Correspondence: dr_morenheim@hotmail.com; Tel.: +52-999-942-7600 (ext. 54304)

Supplementary Table S1. Comparation of General Characteristics of total population and patients with and without COPD after spirometry confirmation.

Characteristics	Total population (n=197)	Without COPD (n=124)	With COPD (n=73)	p
Age, years. Median (IQR)	67 (58, 75)	64 (57, 70)	72 (63, 79)	<0.001
Age, years; frequency (%)				0.177
40- 49	14 (7.1)	11 (8.9)	3 (4.1)	
50- 59	44 (22.3)	31 (25.0)	13 (17.8)	
> 60	139 (70.6)	82 (66.1)	78.1 (78.1)	
Gender, frequency (%)				0.475
Female	82 (41.6)	54 (43.6)	28 (38.4)	
Male	115 (58.4)	70 (56.4)	45 (61.6)	
Comorbidities, frequency (%)	165 (83.8)	165 (83.8)	61 (83.6)	0.955
Systemic hypertension, frequency (%)	90 (45.7)	90 (45.7)	35 (47.9)	0.625
Diabetes Mellitus 2, frequency (%)	54 (27.4)	54 (27.4)	16 (21.9)	0.185
Cardiovascular, frequency (%)	34 (17.3)	18 (14.5)	16 (21.9)	0.184
Asthma, frequency (%)	9 (4.6)	2 (1.6)	7 (9.6)	0.014
Smoking, frequency (%)	180 (91.4)	111 (89.5)	69 (94.5)	0.227
History of smoking, years; Median (IQR)	26 (17, 40)	21 (15, 40)	33 (20, 43)	0.001
Biomass, frequency (%)	28 (14.2)	21 (16.9)	7 (9.6)	0.154
FEV1, %; Median (IQR)	75.0 (60.4, 90.0)	84.0 (73.0, 95.8)	61.0 (47.0, 70.0)	<0.001
FEV1 z-score, Median (IQR)	-1.39 (-2.42, -0.42)	-0.91 (-1.61, 0.0)	-2.33 (-2.92, -1.64)	<0.001
FVC, %; Median (IQR)	81.0 (66.0, 92.0)	82.0 (72.0, 92.4)	78.0 (59.0, 89.0)	0.023
FVC z-score, Median (IQR)	-1.28 (-2.0, -0.5)	-1.25 (-1.85, -0.47)	-1.46 (-2.13, -0.71)	0.188
Relation FEV1/FVC, Median (IQR)	0.73 (0.63, 0.80)	0.78 (0.74, 0.82)	0.60 (0.52, 0.65)	<0.001
PUMA Score	6 (5, 7)	5 (4, 7)	7 (5,8)	<0.001
CAPTURE Score	3 (2, 4)	3 (2, 4)	4 (3,5)	0.009

Table S2. Spearman correlation between PUMA, CAPTURE, and spirometry parameters before PS matching *

VARIABLES	PUMA (n=197)		CAPTURE (n=48)	
	r	P	r	p
FEV1, liters;	-0.316	<0.001	-0.401	0.004
FEV1, %;	-0.321	<0.001	-0.322	0.025
FEV1 z-score,	-0.293	<0.001	-0.582	<0.001
FVC, liters;	-0.181	0.010	-0.279	0.054
FVC, %	-0.219	0.002	-0.517	<0.001
FVC z-score	-0.120	0.091	-0.294	0.042
FEV1/FVC	-0.390	<0.001	-0.406	0.004
z-score FEV1/FVC	-0.269	<0.001	-0.383	0.007

*propensity score matching

Figure S1. Analysis of variables for propensity score matching

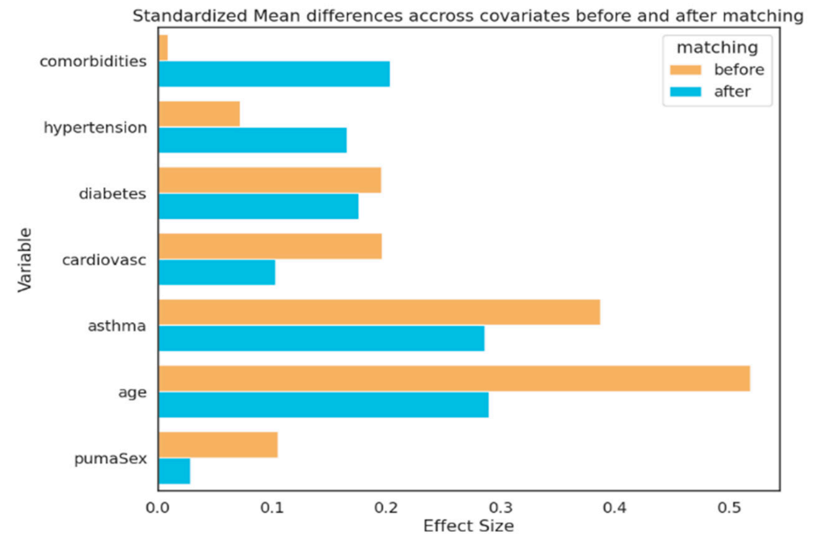

Variable	matching	Effect Size
comorbidities	before	0.008344
comorbidities	after	0.203074
hypertension	before	0.071753
hypertension	after	0.165076
diabetes	before	0.195531
diabetes	after	0.175892
cardiovasc	before	0.195749
cardiovasc	after	0.102433
asthma	before	0.386689
asthma	after	0.285740
age	before	0.517727
age	after	0.289177
pumaSex	before	0.104933
pumaSex	after	0.028079

Table S3. Spearman correlation between PUMA, CAPTURE and spirometry parameters after PS matching*

	PUMA (n=73)		CAPTURE (n=42)	
	r	p	r	p
FEV1, liters;	-0.271	<0.001	-0.393	0.009
FEV1, %;	-0.293	<0.001	-0.338	0.028
FEV1 Z Score,	-0.288	<0.001	-0.619	<0.001
FVC, liters;	-0.133	0.108	-0.240	0.124
FVC, %	-0.198	0.019	-0.540	<0.001
FVC Z Score	-0.113	0.173	-0.281	0.070
FEV1/FVC	-0.384	<0.001	-0.451	0.002
Z Score FEV1/FVC	-0.276	<0.001	-0.436	0.003

*propensity score matching

Table S4. Linear regression to identify the association of the FEV1/FVC index with the PUMA and CAPTURE scores before PS matching*

	PUMA (n=197)		CAPTURE (n=48)	
Characteristics	Coefficient (CI 95%)	p	Coefficient (CI 95%)	p
Score	-0.023 (-0.034, -0.012)	***<0.001***	-0.031 (-0.056, -0.006)	***0.014***
Age, years	-0.002 (-0.003, -0.0005)	***0.010***	-0.002 (-0.006, 0.001)	0.195
Sex	-0.015 (-0.049, 0.019)	0.384	-0.050 (-0.124, 0.024)	0.182
Asthma	-0.104 (-0.108, -0.023)	***0.012***	0.102 (-0.064, 0.270)	0.223
Cardiovascular	0.013 (-0.031, 0.058)	0.546	-0.041 (-0.136, 0.052)	0.378
Diabetes Mellitus	0.036 (-0.002, 0.075)	0.070	0.028 (-0.074, 0.131)	0.578
High blood pressure	-0.027 (-0.064, 0.010)	0.154	0.005 (-0.089, 0.1003)	0.907
Comorbidities	0.014 (-0.035, 0.064)	0.572	-0.044 (0.723, 1.239)	0.455

*propensity score matching

Figure S2. Residual charts to assess linearity and normality for the LRA analysis for PUMA and CAPTURE before PS matching*

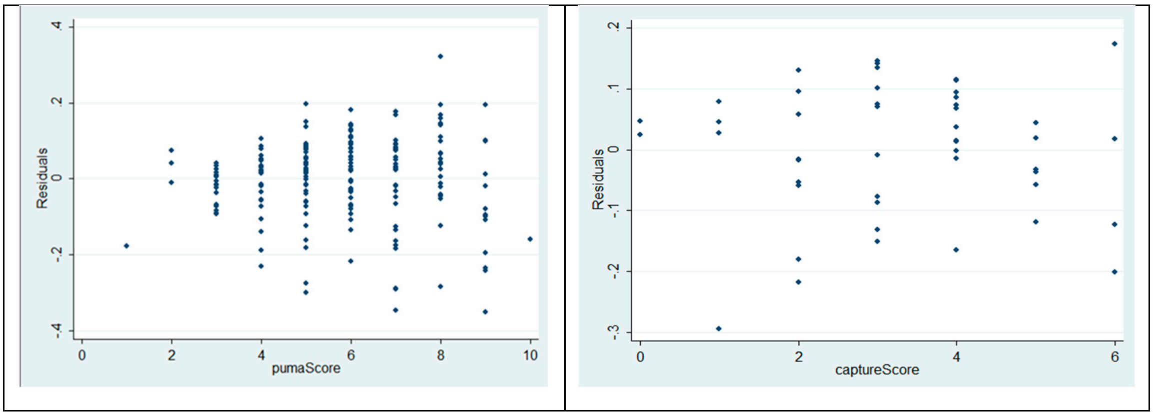

*propensity score matching

Table S5. Linear regression to identify the association of FEV1/FVC index with PUMA and CAPTURE scores after PS matching*

	PUMA (n=73)		CAPTURE (n=42)	
Characteristics	Coefficient (CI 95%)	p	Coefficient (CI 95%)	p
Score	-0.028 (-0.041, -0.015)	***<0.001***	-0.037 (-0.06, -0.010)	***0.009***
Age, years	-0.001 (-0.003, 0.0009)	0.316	-0.001 (-0.006, 0.002)	0.458
Sex	-0.003 (-0.046, 0.040)	0.889	-0.029 (-0.111, 0.052)	0.465
Asthma	-0.084 (-0.175, 0.007)	0.071	0.151 (-0.023, 0.326)	0.087
Cardiovascular	0.021 (-0.032, 0.0754)	0.429	-0.012 (-0.0110, 0.086)	0.802
Diabetes Mellitus	0.009 (-0.047, 0.066)	0.747	-0.004 (-0.122, 0.113)	0.944
High blood pressure	-0.222 (-0.070, 0.0274)	0.378	0.040 (-0.071,0.152)	0.469
Comorbidities	-0.009 (-0.070, 0.050)	0.746	-0.89 (-0.216, 0.038)	0.163

*propensity score matching

Figure S3. Residual charts to assess linearity and normality for the LRA analysis for PUMA and CAPTURE after PS matching*

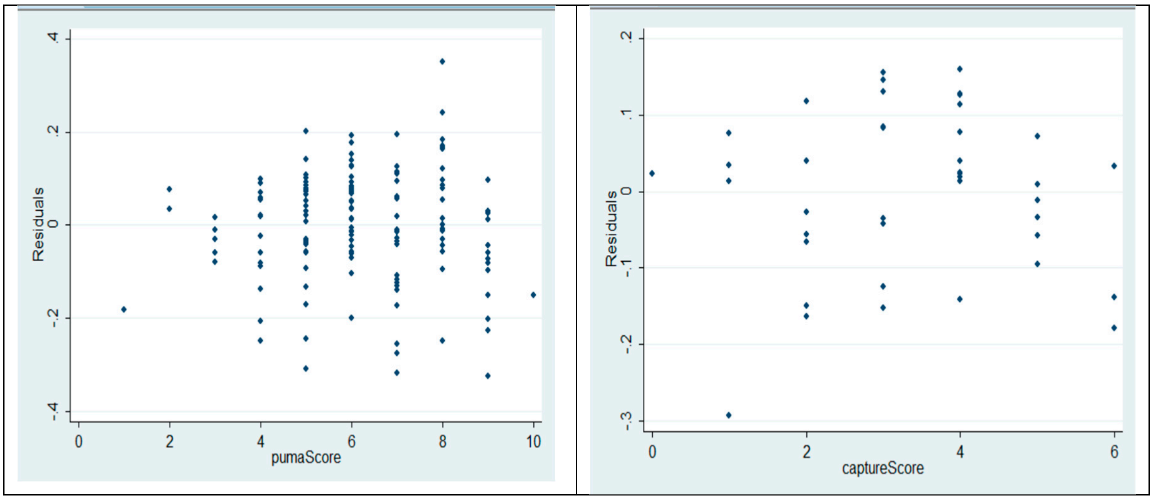

*propensity score matching

Table S6. Sensitivity and specificity of the PUMA questionnaire with different cut-off points (n=197) using pre-BD FEV$_1$/FVC with a fixed cut-off point of 0.70 (70%)

PUMA score	Sensitivity (%)	Specificity (%)	Correct classification (%)	LR+	LR-
≥ 4	85.5	2.7	54.8	0.87	5.29
≥ 5	70.1	10.9	48.2	0.78	2.72
≥ 6	48.4	26.0	40.1	0.65	1.98
≥ 7	25.8	45.2	32.9	0.47	1.64
CAPTURE score					
≥ 2	77.3	3.8	37.5	0.80	5.90
≥ 3	59.1	23.1	39.6	0.76	1.77
≥ 4	27.3	38.4	33.3	0.44	1.89
≥ 5	4.5	65.4	37.5	0.13	1.45

Table S7. Sensitivity and specificity of the PUMA questionnaire with different cut-off points (n=197) using LLN (z-score < -1.645) to define obstruction.

PUMA score	Sensitivity (%)	Specificity (%)	Correct classification (%)	LR+	LR-
≥ 4	98.8	13.0	35.0	1.12	0.15
≥ 5	92.1	28.1	44.6	1.28	0.27
≥ 6	76.5	48.6	55.8	1.48	0.48
≥ 7	60.8	71.9	69.0	2.16	0.54
CAPTURE score					
≥ 2	95.0	17.8	50.0	1.15	0.28
≥ 3	75.0	35.7	52.0	1.16	0.70
≥ 4	55.0	60.7	58.3	1.40	0.74
≥ 5	40.0	92.8	70.8	5.60	0.64
